# COVID‐19 lockdown and poor sleep quality: Not the whole story

**DOI:** 10.1111/jsr.13368

**Published:** 2021-05-06

**Authors:** Valentina Alfonsi, Maurizio Gorgoni, Serena Scarpelli, Pierpaolo Zivi, Stefano Sdoia, Emanuela Mari, Angelo Fraschetti, Fabio Ferlazzo, Anna Maria Giannini, Luigi De Gennaro

**Affiliations:** ^1^ Department of Psychology Sapienza University of Rome Rome Italy; ^2^ IRCCS Fondazione Santa Lucia Rome Italy

**Keywords:** COVID‐19 lockdown, gender bias, sleep components, sleep quality

## Abstract

A growing body of evidence consistently describes the side‐effects of coronavirus disease 2019 lockdown on mental health and sleep quality. We conducted a longitudinal web‐based survey of 217 Italian participants at two time points: lockdown and subsequent follow‐up. To thoroughly investigate lockdown‐related changes in sleep quality, we first evaluated variations in overall sleep quality assessed by the Pittsburgh Sleep Quality Index. We then examined sleep changes in specific components of sleep quality. Results revealed a clear dissociation of sleep effects, as a function of the specific domain considered, with longer sleep latency, worse sleep efficiency, and massive sleep medication use during forced confinement. On the other hand, we simultaneously observed an increased sleep duration and better daytime functioning. Our present findings highlight the importance of an accurate examination of sleep quality during lockdown, as the effects were not uniform across populations and different sleep domains.

## 1 INTRODUCTION

Many countries implemented public health measures to control the rapid worldwide spread of the coronavirus virus disease 2019 (COVID‐19). Italy imposed a total lockdown (09/03/2020–04/05/2020), forcing its inhabitants to stay at home and go out only when strictly needed.

This unprecedented situation caused dramatic changes in social relationships and daily habits, having a substantial impact on physical and mental wellbeing. As expected, people increased their levels of anxiety, stress, and depression, and were more prone to develop severe mental symptomatology (Pfefferbaum & North, 2020; Rajkumar, 2020; Torales, O'Higgins, Castaldelli‐Maia, & Ventriglio, 2020).

Although many studies have consistently shown the huge costs of the COVID‐19 outbreak on psychological health, the effects of restrictions on sleep quality depict a more complex scenario.

As correctly stated in the first months of 2020 (Altena et al., 2020), recent findings showed that forced home confinement affected sleep habits in different ways, resulting in both beneficial and detrimental effects on sleep timing and duration (Casagrande, Favieri, Tambelli, & Forte, 2020; Cellini, Canale, Mioni, & Costa, 2020; Franceschini et al., 2020; Holzinger, Mayer, Nierwetberg, & Klösch, 2021; Kocevska, Blanken, Van Someren, & Rösler, 2020; Wright et al., 2020). Furthermore, gender played a crucial role in mediating the impact of this stressful situation on the overall sleep quality, showing an increased vulnerability in females (Conversano et al., 2020; Salfi, Lauriola, et al., 2020).

Keeping in mind such a heterogeneous framework, we aimed to investigate the effects of restrictive measures on several sleep‐quality domains through a longitudinal study considering both lockdown and post‐lockdown periods. Specifically, we were interested in observing changes (a) in global sleep quality weighting by gender type and (b) in every single component of sleep quality separately. We expected stable worse sleep conditions in females and lockdown‐related mixed effects on sleep quality, depending on the specific domain considered.

## 2 METHODS

### 2.1 Participants and procedure

A longitudinal survey was carried out on adult Italian citizens at two time points: during the fourth week of the Italian lockdown (T1 – lockdown: 29/03/2020–05/04/2020) and 4 months after the end of the national lockdown (T2 – post‐lockdown: 14/09/2020–12/10/2020).

We used a web‐based survey implemented on the Qualtrics Survey Platform and shared via social media, email or telephone.

All individuals explicitly agreed to participate to the research and completed the survey after reading and signing the electronic informed consent. Participants could withdraw from the study at any moment and identification codes were created to anonymise personal data.

The study was conducted in accordance with the Declaration of Helsinki and approved by the Institutional Review Board of the Department of Psychology of the Sapienza University of Rome (Prot. #577, 28 March 2020).

Data reported in this study were part of a wider research longitudinal project concerning the psychological impact of home confinement in Italy, and other data with different purposes will be presented elsewhere.

### 2.2 Measures

#### 2.2.1 Sociodemographic and COVID‐19‐related information

An initial questionnaire was administered to assess sociodemographic characteristics, such as gender, age, education, occupation and marital status. A subsequent set of questions assessed COVID‐19‐related information (e.g. COVID‐19‐infected relatives or friends, forced quarantine period).

#### 2.2.2 Sleep measures

Sleep quality was assessed by the Pittsburgh Sleep Quality Index (PSQI) (Buysse, Reynolds, Monk, Berman, & Kupfer, 1989; Curcio et al., 2013). The PSQI is a well‐known self‐report questionnaire investigating sleep quality over 1 month. It comprises 19 items, from which partial scores in seven subscales (ranging from 0 to 3) and a global score (ranging from 0 to 21) are calculated. The subscales refer to seven different components: subjective sleep quality, sleep latency, sleep duration, sleep efficiency, sleep disturbances, use of sleep medications, and daytime dysfunction. A PSQI global score of >5 indicates poor sleep quality.

### 2.3 Statistical analyses

We performed an exact McNemar’s test to evaluate the variation in the proportion of poor and good sleep during and after the lockdown. Then, we extracted specific information from the PSQI questionnaire, such as bedtime (BT, 24‐hr clock), rise time (RT, 24‐hr clock), and total bedtime (TBT, min). Paired *t* tests were computed to assess pre–post changes in these variables.

According to the purpose of the study, we considered the global PSQI score and all its components as the main variables of interest.

The PSQI global score was analysed in a mixed‐design repeated‐measures analysis of variance (ANOVA), considering Time (lockdown versus post‐lockdown) and Gender (male versus female) as within‐ and between‐subjects factors, respectively. *Post hoc* comparisons were conducted using the Tukey “honestly significant difference” HSD test.

The Wilcoxon signed‐rank test was used to evaluate pre–post changes of sleep dimensions for each PSQI component.

All data were analysed using the Statistical Package for the Social Sciences (SPSS®; version 25.0; IBM SPSS). A *p* ≤ .05 was considered statistically significant.

## 3 RESULTS

### 3.1 Demographic and COVID‐related characteristics

We received 905 completed questionnaires at T1. We excluded four subjects who completed the questionnaire more than once and three non‐Italian subjects. Of the 898 respondents at T1, 217 individuals also took part in the follow‐up phase (mean age [*SD*, range] 35.68 [15.96, 18–75] years, 157 females) and represented our final sample for the subsequent analyses. Demographic and COVID‐related characteristics are shown in Table 1.

**TABLE 1:** Descriptive characteristics of the longitudinal sample (*N* = 217)

Characteristic	*N* (%)
Gender	
Male	60 (28)
Female	157 (72)
Age, years	
18–30	125 (58)
31–50	29 (13)
>50	63 (29)
Education	
Until middle School	17 (8)
High School	94 (43) 107 (49)
Graduate	
Occupation	
Student	91 (42)
Employed	103 (47)
Unemployed or retired	25 (11)
Marital status	
Single	148 (68)
Married	55 (25)
Separated/divorced	14 (7)
COVID‐19‐related features	
Infected relative/friend	
Yes	19 (9)
No	198 (91)
Forced quarantine	
Yes	3 (1)
No	214 (99)

### 3.2 Longitudinal changes in sleep quality

During lockdown, the proportion of good and poor sleepers was almost equivalent, with a slightly higher prevalence of poor sleepers (49% versus 51%). Conversely, the increased number of good sleepers along with the decreased number of poor sleepers describes an opposite pattern in the follow‐up assessment (56% versus 44%; McNemar’s test: *p* = .05).

**FIGURE 1:**
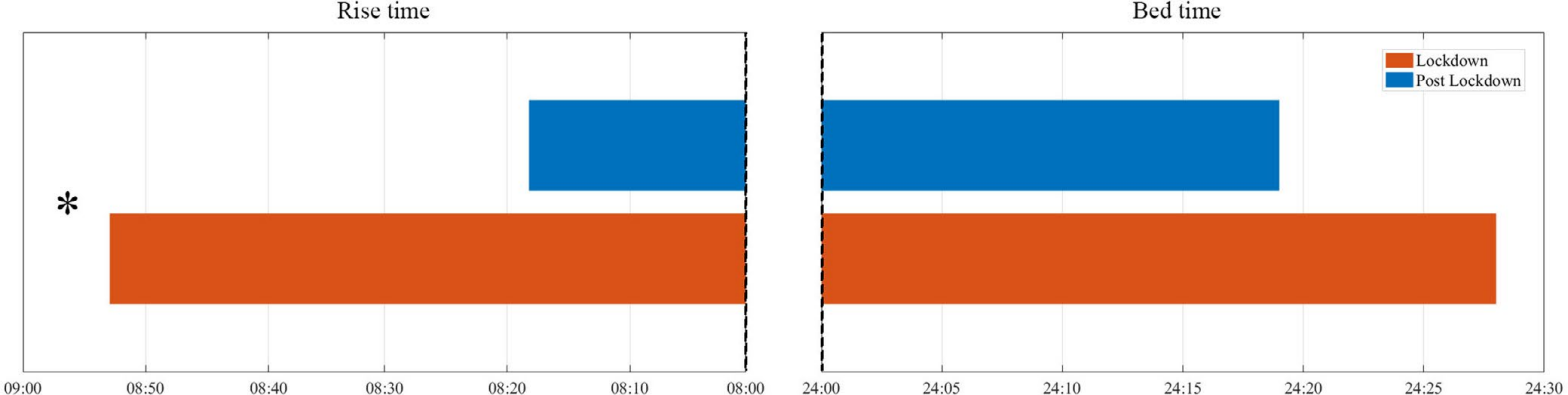
Rise time (24‐hr clock) and bedtime (24‐hr clock) of participants during lockdown and post‐lockdown. *Significant at *p* ≤ .05

The lockdown mostly affected the rise time and the time spent in bed, with a smaller effect on the bedtime. The results showed that during lockdown subjects woke‐up later (mean [*SE*] T1 = 08:53 [0.108] hr, T2 = 08:18 [0.110] hr; *t*_216_ = 5.89, *p* < .001) and spent more time in bed (mean [*SE*] T1 = 509.66 [5.879] min, T2 = 478.56 [5.347] min; *t*_216_ = 4.55, *p* < .001), while bedtime only approached to significance (mean [*SE*] T1 = 24:28 [0.104] hr, T2 = 24:19 [0.103] hr; *t*_216_ = 1.69, *p* = .093) (Figure 1).

​

​

**FIGURE 2:**
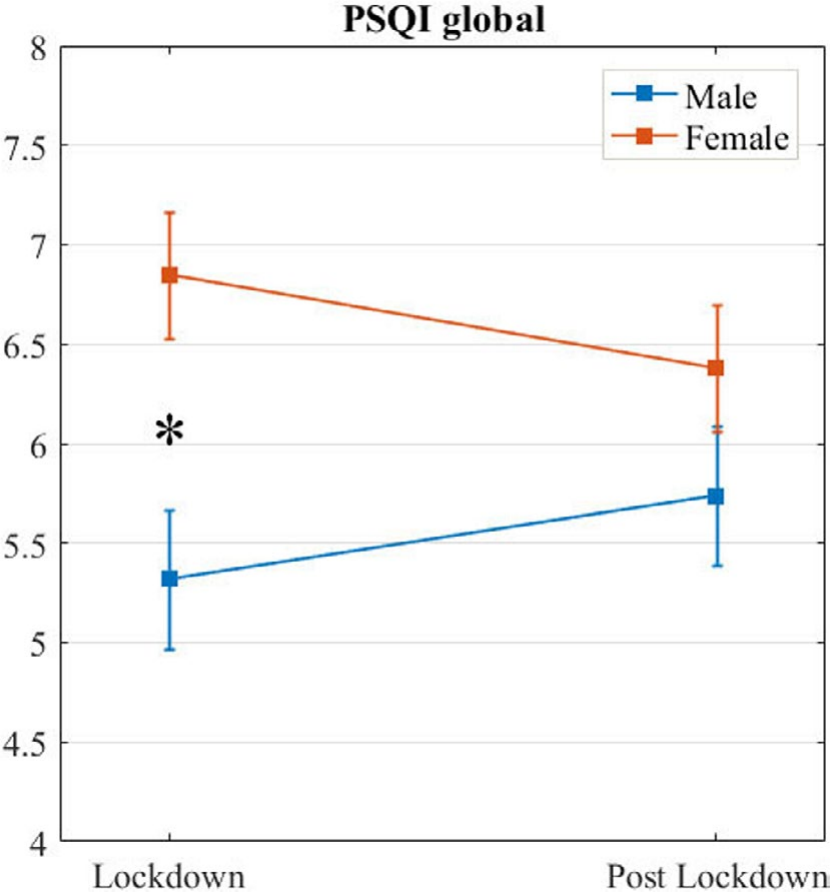
Mean (*SD*) global Pittsburgh Sleep Quality Index (PSQI) scores for the two time points (lockdown and post‐lockdown) in males and females. *Significant at *p* ≤ .05

The repeated‐measures ANOVA time × gender on PSQI global scores (Figure 2) showed a significant main effect of gender (*F*_1,215_ = 5.95, *p* = .016) and a significant interaction between time and gender (*F*_1,215_ = 4.17, *p* = .042), reflecting higher PSQI global scores in females compared to males in lockdown (*t* = −3.09, p_tukey_ = .012), and no significant differences between genders in post‐lockdown (*t* = −1.28, p_tukey_ = .575). No significant main effect of time was found (*F*_1,215_ = 0.02, *p* = .889).

Concerning the pre–post changes for each PSQI component, Table 2 depicts the results of the comparisons. We observed a significant lockdown‐related worsening (as reflected by higher PSQI scores) of sleep latency (*Z* = −3.517, *p* ≤ .001), sleep efficiency (*Z* = −2.787, *p* = .005) and use of sleep medication (*Z* = −2.291, *p* = .022), and a significant improvement (as reflected by lower PSQI scores) in sleep duration (*Z* = 2.736, *p* = .006) and daytime dysfunction (*Z* = 3.679, *p* ≤ .001).

**TABLE 2:** Pre–post changes (Wilcoxon signed‐rank test) in each Pittsburgh Sleep Quality Index (PSQI) component.

PSQI component	Lockdown	Post‐lockdown	*Z*	*p*
	Mean (*SE*)	Mean (*SE*)		
Subjective sleep quality	1.212 (0.05)	1.189 (0.04)	−0.394	.693
Sleep latency	1.267 (0.06)	1.037 (0.06)	−3.517	**<.001**
Sleep duration	0.866 (0.05)	1.028 (0.05)	2.736	.**006**
Sleep efficiency	0.728 (0.07)	0.521 (0.05)	−2.787	.**005**
Sleep disturbances	1.175 (0.03)	1.170 (0.03)	−0.109	.913
Use of sleep medications	0.258 (0.05)	0.138 (0.03)	−2.291	.**022**
Daytime dysfunction	0.682 (0.04)	0.876 (0.05)	3.679	**<.001**

*SE*, standard error. Bold values denote statistical significance at *p* ≤ .05

No difference was found between lockdown and post‐lockdown with regard to subjective sleep quality (*Z* = −0.394, *p* = .693) and sleep disturbances (*Z* = −0.109, *p* = .913).

## 4 DISCUSSION

To the best of our knowledge, this is the first longitudinal study designed to understand the impact of restriction measures on sleep quality, taking into account all the variables of the PSQI (global and single components) and considering both the lockdown period and the subsequent follow‐up measurements.

Under daily routine conditions, epidemiological studies among the general population reported a prevalence rate of poor sleepers ranging between 30% and 40% (Hinz et al., 2017). In keeping with the studies describing the worsening of sleep quality during the COVID‐19 pandemic (Jahrami et al., 2021), our present findings identified an increase in poor sleepers during the lockdown (51%) and a partial restoration of the normative prevalence of good and poor sleepers in the follow‐up phase (56% and 44%, respectively).

Consistent with previous literature (Hinz et al., 2017), females had higher PSQI global scores, regardless of the time points. Additionally, this gender gap was amplified during the lockdown, as demonstrated by a significant difference in this period with respect to the post‐quarantine period. As stated in previous studies, additional lockdown‐related stress, such as taking care of children and other family duties, could further exacerbate women’s probability of developing sleep problems (Conversano et al., 2020; Salfi, Lauriola, et al., 2020). The greater stressors endured by women during the lockdown were also reflected by an increased prevalence of negative emotions and contents in female dreams (Kilius, Abbas, McKinnon, & Samson, 2021). Further, men tend to be later chronotypes than women (Randler, 2007), which could explain the greater benefits from more flexible wake‐up times in the male gender.

Given the multi‐components nature of the PSQI global score and the mounting evidence of both beneficial and detrimental effects on sleep timing and duration, we focussed on the specific components of the PSQI. In this regard, results revealed a clear dissociation of sleep effects as a function of the specific domain considered.

We observed longer sleep latency, worse sleep efficiency, and massive sleep medication use during forced confinement. On the other hand, we also observed an increased sleep duration and better daytime functioning during the lockdown. Clearly, such dissociation between worsening and improving aspects explains the absence of pre–post changes in the global PSQI score. Furthermore, it highlights that the evident costs of living under restrictions need to be carefully weighed against the possible benefits on sleep patterns resulting from this extraordinary situation. For instance, as a consequence of smart working or home schooling, people had the opportunity to wake‐up later (08:53 versus 08:18 hours), which could explain the increased time spent in bed (510 versus 479 min). However, this increase may not be paralleled by the same extension in actual sleeping time, resulting in reduced sleep efficiency. Moreover, the reduced impact of social zeitgebers (e.g. work or school schedules) could have determined the individuals’ chance to set bedtime and rise time in accordance with their circadian preference (Korman et al., 2020), and this may be related to the perceived better diurnal performance. Another phenomenon became relevant during the confinement, namely the increase of digital media use near bedtime (Salfi, Amicucci, et al., 2020). Evening screen exposure may further exacerbate the subjective difficulty in falling asleep, reflected in the lengthening of the sleep onset latency and, indirectly, in the massive use of hypnotics (Beck, Léger, Fressard, Peretti ‐ Watel, & Verger, 2020). Not surprisingly, the side‐effects on sleep were also closely related to the alarming spread of psychological distress during the pandemic (Cellini et al., 2020; Franceschini et al., 2020; Zhang, Zhang, Ma, & Di, 2020).

The abrupt change of living conditions involved many other stay‐at‐home circumstances (e.g. reduced daylight exposure, limited physical activity, change in meal times, etc.), influencing sleep quality differently. Hence, we suggest that (a) the consequences of this unexpected context on sleep are wide‐ranging and not fully represented by the composite nature of the global PSQI score; (b) a proper evaluation of the impact of lockdown on sleep should simultaneously consider both risk and protective factors arising from this extreme situation, along with individual vulnerability.

To sum up, our present results offer a possible explanation about the divergent findings in the existing literature, pointing out the manifold nature of the effects of home confinement on sleep habits. However, caution is needed in interpreting the results, considering both the lack of pre‐quarantine measures as a baseline and the self‐selection bias linked to the online recruitment strategy. We should also keep in mind that even if restrictive measures were partially mitigated during the post‐lockdown period, daily lives and sleeping patterns were not the same as before the pandemic, and this might affect the sleep‐quality rating.

Given the bidirectional relationship between sleep quality and the psychological and emotional impact of the COVID‐19 outbreak (Zhang et al., 2020), the influence of lockdown on sleep characteristics should be analysed carefully. Especially, an effort should be made to define the specific domains of sleep quality negatively affected by the COVID‐19‐associated events, with the ultimate goal to avoid incorrect generalisations and efficiently deal with these unprecedented changes in sleep habits.

## CONFLICT OF INTEREST

None of the authors have potential conflicts of interest to be disclosed.

## AUTHOR CONTRIBUTIONS

VA, MG, FF, AMG and LDG conceived and designed the experiment; SSc, PZ, SSd, EM, AF collected and pre‐processed the data; VA and MG analysed the data; VA, MG and LDG wrote the paper.

## Data Availability

The data that support the findings of this study are available from the corresponding author upon reasonable request.
